# Isolation of SARS-CoV-2 in Viral Cell Culture in Immunocompromised Patients With Persistently Positive RT-PCR Results

**DOI:** 10.3389/fcimb.2022.804175

**Published:** 2022-02-02

**Authors:** Abby Sung, Adam L. Bailey, Henry B. Stewart, David McDonald, Meghan A. Wallace, Kate Peacock, Candace Miller, Kimberly A. Reske, Caroline A. O’Neil, Victoria J. Fraser, Michael S. Diamond, Carey-Ann D. Burnham, Hilary M. Babcock, Jennie H. Kwon

**Affiliations:** ^1^ Department of Medicine, Division of Infectious Diseases, Washington University School of Medicine in St. Louis, St. Louis, MO, United States; ^2^ Department of Pathology and Immunology, Washington University School of Medicine in St. Louis, St. Louis, MO, United States; ^3^ Department of Molecular Microbiology, Washington University School of Medicine in St. Louis, St. Louis, MO, United States; ^4^ Departments of Medicine and Pediatrics, Washington University School of Medicine in St. Louis, St. Louis, MO, United States

**Keywords:** COVID-19, SARS-CoV-2, immunocompromised, rituximab, obinutuzumab, laboratory medicine, viral cell culture

## Abstract

Immunocompromised adults can have prolonged acute respiratory syndrome coronavirus 2 (SARS-CoV-2) positive RT-PCR results, long after the initial diagnosis of coronavirus disease 2019 (COVID-19). This study aimed to determine if SARS-CoV-2 virus can be recovered in viral cell culture from immunocompromised adults with persistently positive SARS-CoV-2 RT-PCR tests. We obtained 20 remnant SARS-CoV-2 PCR positive nasopharyngeal swabs from 20 immunocompromised adults with a positive RT-PCR test ≥14 days after the initial positive test. The patients’ 2^nd^ test samples underwent SARS-CoV-2 antigen testing, and culture with Vero-hACE2-TMPRSS2 cells. Viral RNA and cultivable virus were recovered from the cultured cells after qRT-PCR and plaque assays. Of 20 patients, 10 (50%) had a solid organ transplant and 5 (25%) had a hematologic malignancy. For most patients, RT-PCR Ct values increased over time. There were 2 patients with positive viral cell cultures; one patient had chronic lymphocytic leukemia treated with venetoclax and obinutuzumab who had a low viral titer of 27 PFU/mL. The second patient had marginal zone lymphoma treated with bendamustine and rituximab who had a high viral titer of 2 x 10^6^ PFU/mL. Most samples collected ≥7 days after an initial positive SARS-CoV-2 RT-PCR had negative viral cell cultures. The 2 patients with positive viral cell cultures had hematologic malignancies treated with chemotherapy and B cell depleting therapy. One patient had a high concentration titer of cultivable virus. Further data are needed to determine risk factors for persistent viral shedding and methods to prevent SARS-CoV-2 transmission from immunocompromised hosts.

## Introduction

Coronavirus disease 2019 (COVID-19) was declared a public health emergency by the World Health Organization (WHO) in January 2020 and deemed a global pandemic in March 2020. Detection of viral RNA using real time, reverse transcriptase-polymerase chain reaction (RT-PCR) is the gold standard diagnostic test for SARS-CoV-2, the virus that causes COVID-19. Many patients diagnosed with COVID-19 have multiple positive repeat RT-PCR tests for SARS-CoV-2 even after resolution of COVID-19 symptoms (Lu et al., 2020; Owusu et al., 2021). Although replication-competent virus has not been isolated from most immunocompetent patients with mild symptoms after 10 days, there have been reports of persistent shedding of replication-competent, cell culture positive SARS-CoV-2 virus in immunocompromised hosts beyond 20 days (Avanzato et al., 2020; Aydillo et al., 2020; Choi et al., 2020; Kim et al., 2021).

Currently, CDC recommendations acknowledge that severely immunocompromised patients may have persistent viral shedding beyond 20 days, thus recommending consideration of infectious disease consultation and additional testing before removing isolation precautions (“Ending Isolation and Precautions for People with COVID-19: Interim Guidance,” 2021). Whether or not and how long immunocompromised patients shed infectious virus has profound implications for understanding disease transmission and treatment for these patients. This study aimed to determine if SARS-CoV-2 virus could be recovered in viral cell culture from immunocompromised adults with a repeat positive SARS-CoV-2 RT-PCR tests ≥14 days after their first positive RT-PCR test.

## Methods

### Study Design and Patient Identification

This was a single-center, retrospective, pilot study conducted at Barnes Jewish Hospital (BJH), a large, academic, tertiary referral hospital in Saint Louis, MO.

The BJH medical informatics database was queried to identify adults ≥18 years old with ≥2 positive SARS-CoV-2 RT-PCR tests between March and December 2020. Patients were considered to have a persistently positive result if they had a repeat positive test ≥14 days after their first positive test. Chart review was performed to identify which patients with ≥2 positive SARS-CoV-2 RT-PCR tests were immunocompromised (current cancer treatment, bone marrow or solid organ transplantation, immune deficiencies, HIV with low CD4 count or not on treatment, prolonged use of corticosteroids or other immunosuppressive medications). Remnant NP swabs from the patients’ 2^nd^ test, which was not necessarily the RT-PCR test that qualified the patient for inclusion in the study, were collected from the BJH clinical microbiology laboratory; patients were excluded if remnant NP swab samples were unavailable. The NP swabs were all originally obtained during routine clinical care and included outpatient, inpatient, and emergency room clinical settings at various locations across the BJH system. A convenience sample of 20 immunocompromised patients with eligible NP swabs were included in the analyses. These NP swab samples were sent for antigen testing and viral cell culture.

Additional chart review was performed to collect patient demographic data, clinical characteristics including comorbidities, immunosuppressive medications, and duration and severity of COVID-19 clinical illness.

### Molecular Detection of SARS-CoV-2

For initial clinical RT-PCR testing, acceptable transport tubes for the NP swabs included Universal Transport Medium (UTM) and ESwab (with Aimes Transport Medium). Molecular testing was performed by the BJH Clinical Laboratory and due to supply chain issues and need for high testing volume, multiple assays under Emergency Use Authorization by the FDA were utilized (Raju et al., 2021). Systems utilized included the: Roche *cobas* SARS-CoV-2 assay on the Roche *cobas* 6800 (Roche Molecular Systems, Branchburg, NJ), Cepheid Xpert Xpress SARS-CoV-2 assay on the GeneXpert (Cepheid, Sunnyvale, CA), BioFire Respiratory Panel 2.1 (BioFire, Salt Lake City, UT), DiaSorin Molecular Simplexa™ on the Liaison^®^ MDX (DiaSorin, Saluggia, Italy), and Lyra SARS-CoV-2 Assay (Quidel) on EasyMag(bioMerieux)/RotorGene Q (Qiagen) or KingFisher (Thermofisher)/Applied Biosystems 7500 Fast Dx (Thermofisher). PCR cycle time (Ct) thresholds were recorded for all SARS-CoV-2 RT-PCR tests that were performed by the BJH Clinical Laboratory if available. For tests that provided 2 values, the lower value was used if there was a difference of ≤3 between the 2 values; if one of the values was 0, the larger number was used. As the Lyra assay does not include the first 10 cycles in the reported Ct value, a correction factor of 10 was added to all Ct values provided by the Lyra assay (Ransom et al., 2020; Potter et al., 2021).

### Antigen Testing

The eluate from the NP swab sample (that had been stored frozen at -80°C) from the patients’ remnant second positive NP swab sample was sent for antigen testing on the BD Veritor System (BD, Franklin Lakes, NJ). Samples were vortexed for at least 10 seconds and then a swab from the antigen testing kit was placed in the UTM or ESwab solution. The swab was placed in the reaction tube and swirled in the fluid for at least 15 seconds. Analysis was performed according to the manufacturer instructions for use and the assay interpretation was obtained from the BD Veritor analyzer.

### Viral Cell Culture

#### Biosafety

For the viral culture, all aspects of this study were approved by the office of Environmental Health and Safety at Washington University School of Medicine prior to the initiation of this study. Work with SARS-CoV-2 was performed in a BSL-3 laboratory by vaccinated personnel equipped with powered air purifying respirators.

### Virus Outgrowth Assay

Patient samples were thawed and 250 µL from each sample was mixed with 2.75 mL of Dulbecco’s Modified Eagle Medium (DMEM) containing 5% heat-inactivated fetal bovine serum (FBS), 10 mM HEPES, 1X Penicillin/Streptomycin, and glutamine and amphotericin-B, then passed through a 0.45 µm filter. Media-only was used as negative control and media spiked with 1,000, 100, or 10 PFU of the 2019n-CoV/USA_WA1/2019 SARS-CoV-2 isolate were used as positive controls. Filtered samples were inoculated into T-25 flasks containing Vero cells ectopically expressing TMPRSS2 and human ACE2 (provided by Adrian Creanga and Barney Graham, Vaccine Research Center, NIH) in 5 mL of cell culture media (VanBlargan et al., 2021). Cultures were observed daily for 7 days for cytopathic effect (CPE); cultures that displayed CPE that was inconsistent with typical SARS-CoV-2 were passaged forward, and the original samples were re-filtered and re-inoculated using only 10 µL to reduce cytotoxicity but maximize viral recovery. Upon observation of syncytia formation characteristic of SARS-CoV-2 CPE, culture supernatants were clarified and frozen.

### Viral Sequencing

Viral sequencing was performed at the Washington University McDonnell Genome Institute (Saint Louis, MO). RNA was extracted from cell-culture supernatant using the MagMax Viral 96 kit (ABI) on the Flex System (KingFisher). Extracted RNA was subjected to the ARTIC deep-sequencing protocol which was performed on a HiSeq platform (Illumina) (doi:10.17504/protocols.io.bgxjjxkn).

### Plaque Forming Assay

Vero-TMPRSS2-ACE2 cells (2.5 x 10^5^ cells per well) were added to flat-bottom 12-well tissue culture plates. The following day, media was removed and replaced with 200 µL of 10-fold serial dilutions of the original patient sample in DMEM supplemented with 2% FBS. One hour later, 1 mL of methylcellulose overlay was added. Plates were incubated for 48 hours, then fixed with 4% paraformaldehyde (final concentration) in PBS for 20 minutes. Plates were stained with 0.05% (w/v) crystal violet in 20% methanol and washed twice with distilled, deionized H_2_O.

### Statistics

Descriptive statistics were used to describe patient demographics and clinical characteristics. PCR Ct values were plotted against the time since the first positive RT-PCR test. Analyses were performed using SAS 9.4 (SAS Institute, Cary, NC).

## Results

### Demographics

From 183 patient charts chosen for screening, 36 immuno-compromised patients were identified for further chart review until 20 patients with repeat positive tests with available NP swab specimens were identified for inclusion in the study. From April through December 2020, the lab performed 213,940 COVID-19 tests (including antigen and RT-PCR tests) with 18,165 positive tests. In April, November, and December 2020 the positivity rates were around 14-16% and 3-7% in the other months. Demographic and clinical characteristics are presented in **Table 1**. Median age at the date of the first positive RT-PCR test was 64 years. Ten (50%) of the patients had received a SOT (7 with lung transplants); 5 (25%) patients had a hematologic malignancy, including 1 (5%) patient who had received a bone marrow transplant within 6 months prior to their COVID-19 diagnosis; and 3 (15%) patients had another condition treated with immunosuppressive medication including 1 patient with rheumatoid arthritis, 1 patient with polymyositis, and 1 patient on prednisone for COPD.

**Table 1:** Patient demographics.

Variable	All patients (N=20)
	N (%) or Median (range)
Sex	
Male	11 (55)
Race^a^	
White	14 (70)
Black	6 (30)
BMI	27.2 (20.1 – 52.0)
Age at date of first positive PCR	64 (20 – 79)
Time between first and second positive PCRs (days)	21 (7 – 62)
Number of positive PCR tests after the initial positive test	2 (1-7)
Immunosuppressive condition^b^	
Solid organ transplant	10 (50)
Hematologic malignancy	5 (25)
Bone marrow transplant 6 months beforefirst positive PCR	1 (5)
Other	3 (15)
Solid organ malignancy^b^	1 (5)
Immunosuppressive medication^c^	
Receiving high dose steroids at time of positive PCR test	5 (25)
Receiving biologic medication in prior 30 days	2 (10)
Receiving other immunosuppressive medication in prior 30 days	11 (55)
Other comorbidities	
Hypertension	13 (65)
Heart disease^d^	12 (60)
Chronic kidney disease	10 (50)
Dialysis	3 (15)
Chronic lung disease	7 (35)
Chronic obstructive pulmonary disease	4 (20)
Diabetes	6 (30)
Obesity	6 (30)
Current smoker	2 (10)
Chronic liver disease	1 (5)

a All patients were non-Hispanic.

b The patient qualified for the study as they were on dexamethasone for >30 days prior to the first positive RT-PCR test.

c Prednisone status unknown for 1 patient; autoimmune diseases status unknown for one patient.

d Heart failure, coronary artery disease, congenital heart disease, cardiomyopathies, pulmonary hypertension.

### SARS-CoV-2 Molecular Testing, Antigen Testing, and Viral Cell Culture

**Figure 1:**
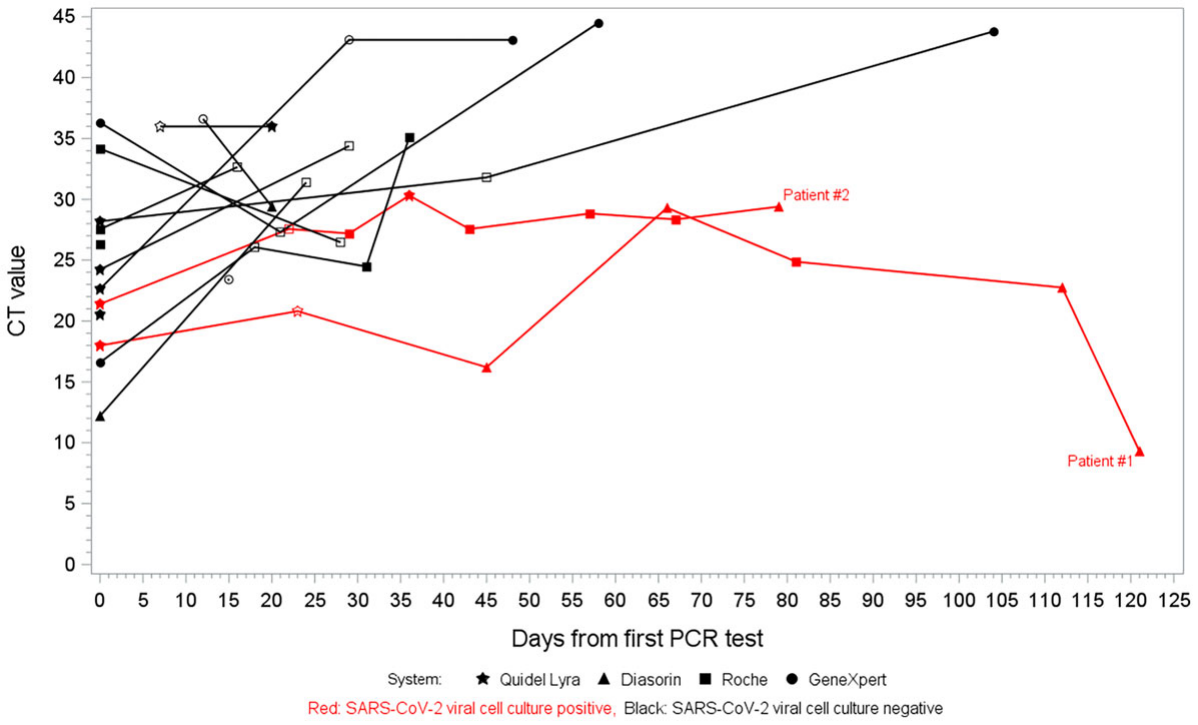
Open symbol indicates sample was cultured.

The median number of RT-PCR tests after the initial positive test was 2 (range 1-7). The median time between the first and second positive RT-PCR tests was 21 days (range 7-62). PCR Ct values for 15 of the 20 patients are shown in **Figure 1**; 5 patients had RT-PCR tests performed on the BioFire assay, which does not provide a Ct value, or had missing Ct values and were therefore not included in the figure. There were 8 patients with increasing Ct values over time, 3 patients with decreasing Ct values, and in 4 patients, the Ct value remained stable or only had one value recorded. However, one patient (#1) had Ct values that initially increased before significantly decreasing on subsequent tests.

Of the 20 samples tested, antigen testing was positive in 6 (30%). There were 2 samples that yielded positive viral cell cultures (**Table 2**). Both patients had hematologic malignancies and were being actively treated with chemotherapy and an anti-CD20 monoclonal antibody with associated hypogammaglobulinemia.

**Table 2:** Characteristics of patients with a positive SARS-CoV-2 cell culture.

Variable	Patient #1	Patient #2
History at time of first positive SARS-CoV-2 RT-PCR	60 year old male with chronic lymphocytic leukemia who presented with cough and diarrhea.	75 year old male with marginal zone lymphoma who presented with 2 weeks of cough.
Other medical conditions	Fibromyalgia	Hyperlipidemia
	Hyperlipidemia	Deep vein thrombosis
		Acute hemolytic anemia
Positive SARS-CoV-2 RT-PCR tests (days after first positive test Day 0) (study samples in **bold**)	Day 0	Day 0
	Day 81	Day 57
	**Day 23**	**Day 22**
	Day 111	Day 67
	Day 45	Day 29
	Day 120	Day 79
	Day 66	Day 36
		Day 43
Malignancy treatment (last dose prior to positive test)	Obinutuzumab and venetoclax (Day -19)	Bendamustine and rituximab (Day -11)
SARS-CoV-2 antigen test	Positive	Negative
Cause of death	COVID-19	Alive as of 16 months after COVID-19 diagnosis
SARS-CoV-2 cell culture results from the repeat test (plaque forming units/mL)	27 PFU/mL	2 x 10^6^ PFU/mL
Spike protein mutations from the repeat test	D614G	D614G, S98F, S813l

### Patient Characteristics

Patient #1 was a 60 year-old man with chronic lymphocytic leukemia (CLL) undergoing treatment with venetoclax chemotherapy and obinutuzumab (an anti-CD20 monoclonal antibody). He started this treatment regimen about 5 months prior to his COVID-19 diagnosis, with the last dose of obinutuzumab received 19 days prior to his first positive SARS-CoV-2 RT-PCR test. He was initially hospitalized for management of diarrhea and tested positive for COVID-19 at admission. He developed severe COVID-19 complicated by septic shock, respiratory failure requiring mechanical ventilation, acute on chronic kidney injury with initiation of dialysis in the intensive care unit, and ultimately died from COVID-19 at 18 weeks after diagnosis. Over a span of 4 months, he had 7 positive SARS-CoV-2 RT-PCR tests. Between the 3^rd^ test (day 45 from first positive test) and 4^th^ test (day 66), the Ct values increased. However, the Ct values subsequently decreased. Viral cell culture from the day 23 sample was positive at 27 plaque forming units (PFU)/mL; he also had a positive antigen test at this time.

Patient #2 was a 75 year-old man with marginal zone lymphoma undergoing treatment with bendamustine chemotherapy and rituximab (an anti-CD20 monoclonal antibody). He started this treatment regimen about 3 months prior to COVID-19 diagnosis, with the last dose of rituximab received 11 days prior to his first positive RT-PCR test. He was hospitalized for 4 days and discharged on home oxygen therapy with daily telephone follow-up. He reported persistent fever and shortness of breath, and his symptoms resolved around 8 weeks after his initial COVID-19 diagnosis. Over about 3 months, he had 8 positive RT-PCR tests. On initial repeat testing, the Ct values increased and subsequently stabilized. The viral cell culture from the day 22 sample was positive at 2 x 10^6^ PFU/mL. Notably, he had a negative antigen test at this time.

There were 2 patients with negative SARS-CoV-2 viral cell cultures who were also being treated with rituximab around the time of their first positive SARS-CoV-2 RT PCR test. One patient was a 38 year-old man with polymyositis on high dose steroids and rituximab who presented with fevers and shortness of breath; he last received rituximab 4 months prior to his first positive RT-PCR test. He underwent a 3^rd^ RT-PCR test 4 weeks after the second positive test due to persistent respiratory symptoms; his symptoms and progressive fibrotic pulmonary changes were ultimately attributed to pulmonary complications of polymyositis. The other patient was a 77 year-old woman with CLL on venetoclax and rituximab; she received rituximab 8 days before her first positive RT-PCR test. She initially presented with fevers, shortness of breath with activity, and fatigue. Her shortness of breath lasted for 6 weeks and she had a relatively mild clinical course. She continued to have fatigue afterwards but did not have any additional repeat RT-PCR tests after the second test.

## Discussion

In this pilot study of 20 immunocompromised adults with persistently positive SARS-CoV-2 RT-PCR tests, only 2 patients had positive viral cell cultures detected from their 2^nd^ positive test. Antigen testing of the repeat sample was positive for 6 patients.

Many case reports of immunocompromised patients with persistent COVID-19 have suggested that patients with hematologic malignancy and treatment with B-cell depleting therapy complicated by hypogammaglobulinemia are at particular risk for persistent viral shedding and severe COVID-19 disease (Avanzato et al., 2020; Choi et al., 2020; Fürstenau et al., 2020). Consistent with this, the 2 patients with positive viral cell cultures in our cohort also had hematologic malignancies and were undergoing treatment with chemotherapy and either rituximab or obinutuzumab, both of which are monoclonal antibodies against CD20 which deplete B cells. There were also 2 patients in this study who, despite being on rituximab, had negative viral cell cultures. This is important to note as it has potential implications for viral transmissibility, management, and infection prevention measures. However, despite this need to understand which immunocompromised patients are at risk for continued viral shedding, there are no routinely available tests for use in a real-time, clinical setting to confirm whether a patient has persistent shedding of infectious virus.

SARS-CoV-2 RT-PCR tests are reported as positive or negative, and Ct values are not reported in routine clinical practice. Higher Ct values have been associated with lower viral RNA concentration in a sample load, although the use of the Ct thresholds as a clinical tool has been somewhat controversial. Previous data from outpatient settings demonstrated that Ct values were similar among symptomatic and asymptomatic patients with COVID-19 (Lee et al., 2020), but in hospitalized patients, high viral loads based on lower Ct values at admission were associated with increased mortality (Magleby et al., 2020; Westblade et al., 2020). An association between Ct value and culture positivity has also been previously observed, with Ct values of 13-15 reported from the EZ1 Virus Mini Kit 2.0, which targets the E-gene, being highly correlated with positive viral cell cultures and a Ct value ≥34 associated with negative viral cell cultures (La Scola et al., 2020). Although Ct values have been shown to correlate with severity of SARS-CoV-2 infection and have been used as a surrogate marker for viral load for research purposes, there are many different RT-PCR assays with different gene targets, Ct values differ between testing platforms, and there are no standardized cutoff thresholds for clinical interpretation (Potter et al., 2021; Rhoads et al., 2021).

The use of Ct values as a tool for COVID-19 infection prevention purposes has been investigated previously (Bullard et al., 2020; He et al., 2020). This is of particular interest in immunocompromised patients as SARS-CoV-2 viral load and transmission are likely to be more unpredictable than in immunocompetent patients, and this may have implications with regards to isolation precautions. Recognizing that caution must be exercised in interpretation of Ct values to make clinical inferences, we observed that, for the most part, Ct values increased with subsequent tests, which is what would be expected during the clinical course of COVID-19 and resolution. However, this was not the case for the 2 patients with positive viral cell cultures weeks after their initial positive RT-PCR test.

Patient #1 ultimately developed severe COVID-19 and had multiple RT-PCR tests from the same testing platform showing a significant decrease in Ct values (greater viral RNA levels) while he was clinically decompensating. His viral cell culture had a low titer of 27 PFU/mL, but the sample was collected fairly early in his clinical course at 23 days after his initial COVID-19 diagnosis, and we did not test the subsequent samples to assess the trend in viral load after this time. Antigen testing was positive in this patient from the same sample collected at 23 days after the initial positive RT-PCR test. In contrast, patient #2 had more moderate disease but unexpectedly had a very high viral titer of 2 x 10^6^ PFU/mL from a sample obtained 22 days after his initial COVID-19 diagnosis with a negative antigen test. Although he initially required a short hospitalization of 4 days, he was primarily managed as an outpatient on home oxygen for most of his prolonged 2-month clinical course. He had an initial increase in Ct values as expected, but the Ct values subsequently remained stable until testing was stopped after his first negative test at 93 days after his first positive RT-PCR test.

In this study, only 6 out of 20 patients had a positive antigen test on the repeat sample. Notably, the two patients with positive viral cell cultures both had negative antigen tests. Although the antigen tests have very good specificity near 95-100%, they have lower analytical sensitivity compared to PCR based assays (Scohy et al., 2020; Liu & Rusling, 2021). The BD Veritor system used herein reports a limit of detection of 1.4 x 10^2^ TCID_50_/mL, an estimated clinical sensitivity of 84% with diminished sensitivity after the first five days of symptom onset, and that negative results should be confirmed with a molecular assay (https://www.bd.com/documents/guides/directions-for-use/IDS_BD-Veritor-Plus-SARS-CoV-2-500048916_DF_EN.pdf). Therefore, it is difficult to draw any inferences about the significance of the negative antigen tests from the patients with positive viral cell cultures.

The findings of this study support prior reports showing that immunocompromised patients can have positive SARS-CoV-2 cell cultures beyond 20 days and that patients with hematologic malignancies on B cell depleting therapy seem to be at particularly increased risk (Avanzato et al., 2020; Aydillo et al., 2020; Choi et al., 2020; Fürstenau et al., 2020). We have also shown that the 2 patients we identified with persistent, active COVID-19 with positive viral cell cultures did not have the expected increase in Ct values over multiple repeat RT-PCR tests. Although the use of Ct values is not routine in the clinical management of patients with COVID-19, Ct values may be useful on a case-by-case basis in high-risk patients to make clinical decisions regarding treatment and isolation precautions.

Notably, there were also 2 patients in our cohort treated with rituximab prior to their first positive SARS-CoV-2 RT-PCR test who had negative viral cell cultures from the second positive NP swab sample. Despite having prolonged symptoms, medical records indicate that the treating clinicians thought that these were secondary to the patient’s underlying medical conditions rather than persistent COVID-19 disease and they were not subject to repeat RT-PCR testing because of this.

There were 2 patients who qualified for the study because they had a positive RT-PCR test ≥14 days after the first positive test. However, their 2^nd^ positive tests were at days 7 and 12 respectively and these were the samples that were sent for viral cell culture. Both of these patients had negative viral cell cultures even though this repeat sample was collected <14 days after their first positive test.

When this study was conceived, there were primarily case reports of persistent, cultivable SARS-CoV-2 virus isolated from samples derived from immunocompromised patients. This study used a more systematic approach to assess the question of whether immunocompromised patients might have persistent shedding of potentially infectious virus and included a balanced distribution of patients with immunocompromising conditions of interest (50% with a SOT, 25% with a hematologic malignancy) and thus provides additional clinical insight. There are several limitations to our study. This was a retrospective pilot study with a small sample size utilizing convenience samples. Because we only queried our institutional database for information, we would have missed any additional COVID-19 tests performed by facilities other than the BJH clinical laboratory. Due to supply chain constraints and high volume of testing needed, different SARS-CoV-2 PCR assays were used for diagnosing COVID-19 in the clinical laboratory. Therefore, patients in this study had results reported from different assays, which complicates comparison of Ct values across instruments and genes targeted in the assay (Rhoads et al., 2021). We performed viral cell cultures at a single point and therefore lack longitudinal data about viral load and how it correlated with clinical status and Ct values; this is an important topic for future investigations. This study was performed on samples collected earlier in the pandemic and no known variants of concern were identified; the results may differ now that the predominant strains in the U.S. have changed.

## Conclusion

Our findings indicate that patients with hematologic malignancies on B-cell depleting therapy who develop COVID-19 are at particular risk of having prolonged SARS-CoV-2 viral cell culture positivity. Additional studies should be performed in immunocompromised patients with COVID-19 to further clarify the risk factors and features associated with persistent shedding of potentially infectious SARS-CoV-2. Further study is also needed to determine the best management and infection prevention strategies for these patients.

## Data Availability Statement

The original contributions presented in the study are publicly available. This data can be found here: https://www.ncbi.nlm.nih.gov/bioproject PRJNA780421.

## Ethics Statement 

This study was approved by the Washington University in St. Louis Human Research Protection Office, IRB #202006011. Written informed consent for participation was not required for this study in accordance with the national legislation and the institutional requirements.

## Author Contributions

Study conception and design: JK, HB, and VF. Data acquisition, analysis, and interpretation: AB, HS, DM, MW, KP, AS, JK, HB, C-AB, KR, and CM. Drafting the article: AS, KR, JK, and HB.A.S. wrote the manuscript, with contributions and comments from all authors. All authors contributed to the article and approved the submitted version.

## Funding

This study was supported by the CDC Prevention Epi-Center Grant: 5U54CK000482. JK is supported by the grant 1K23AI137321-01A1 from the National Institute 320 of Allergy and Infectious Diseases. MD is supported by R01 AI157155.

## Conflict of Interest

MD is a consultant for Inbios, Vir Biotechnology, and Carnival Corporation, and on the Scientific Advisory Boards of Moderna and Immunome. The Diamond laboratory has received funding support in sponsored research agreements from Moderna, Vir Biotechnology, and Emergent BioSolutions.

The remaining authors declare that the research was conducted in the absence of any commercial or financial relationships that could be construed as a potential conflict of interest.

## Publisher’s Note

All claims expressed in this article are solely those of the authors and do not necessarily represent those of their affiliated organizations, or those of the publisher, the editors and the reviewers. Any product that may be evaluated in this article, or claim that may be made by its manufacturer, is not guaranteed or endorsed by the publisher.
